# Using Global Positioning Systems (GPS) and temperature data to generate time-activity classifications for estimating personal exposure in air monitoring studies: an automated method

**DOI:** 10.1186/1476-069X-13-33

**Published:** 2014-05-08

**Authors:** Elizabeth Nethery, Gary Mallach, Daniel Rainham, Mark S Goldberg, Amanda J Wheeler

**Affiliations:** 1 Water and Air Quality Bureau, HECSB, Health Canada, 269 Laurier Avenue West, AL 4903C, Ottawa, Ontario K1A 0 K9, Canada; 2 Dalhousie University, Halifax, Nova Scotia, Canada; 3 Department of Medicine, McGill University, Montreal, Canada; 4 Division of Clinical Epidemiology, McGill University Health Center, Montreal, Canada; 5 School of Natural Science, Edith Cowan University, 270 Joondalup Drive, Joondalup, WA 6027, Australia

**Keywords:** Time activity diary, Particulate air pollution, Global Positioning Systems (GPS), Personal exposure

## Abstract

**Background:**

Personal exposure studies of air pollution generally use self-reported diaries to capture individuals’ time-activity data. Enhancements in the accuracy, size, memory and battery life of personal Global Positioning Systems (GPS) units have allowed for higher resolution tracking of study participants’ locations. Improved time-activity classifications combined with personal continuous air pollution sampling can improve assessments of location-related air pollution exposures for health studies.

**Methods:**

Data was collected using a GPS and personal temperature from 54 children with asthma living in Montreal, Canada, who participated in a 10-day personal air pollution exposure study. A method was developed that incorporated personal temperature data and then matched a participant’s position against available spatial data (i.e., road networks) to generate time-activity categories. The diary-based and GPS-generated time-activity categories were compared and combined with continuous personal PM_2.5_ data to assess the impact of exposure misclassification when using diary-based methods.

**Results:**

There was good agreement between the automated method and the diary method; however, the automated method (means: outdoors = 5.1%, indoors other =9.8%) estimated less time spent in some locations compared to the diary method (outdoors = 6.7%, indoors other = 14.4%). Agreement statistics (AC1 = 0.778) suggest ‘good’ agreement between methods over all location categories. However, location categories (Outdoors and Transit) where less time is spent show greater disagreement: e.g., mean time “*Indoors Other*” using the time-activity diary was 14.4% compared to 9.8% using the automated method. While mean daily time “*In Transit*” was relatively consistent between the methods, the mean daily exposure to PM_2.5_ while “*In Transit*” was 15.9 μg/m^3^ using the automated method compared to 6.8 μg/m^3^ using the daily diary.

**Conclusions:**

Mean times spent in different locations as categorized by a GPS-based method were comparable to those from a time-activity diary, but there were differences in estimates of exposure to PM_2.5_ from the two methods. An automated GPS-based time-activity method will reduce participant burden, potentially providing more accurate and unbiased assessments of location. Combined with continuous air measurements, the higher resolution GPS data could present a different and more accurate picture of personal exposures to air pollution.

## Background

An improved ability to capture and classify where people spend their time can help to increase our understanding of exposures and behaviors that may be harmful to human health. There are a wide range of different air pollutants which exist in the human environment and which may impact human health. A pollutant mixture that varies within small spatial distances is urban air pollution. For example, specific traffic-related air pollutants are often higher on busy city streets in comparison to suburban ones [1]. Understanding personal exposure to air pollution is complicated by an individual’s activities and where they are physically located, and the resultant interactions with different levels of air pollution [2-6].

Technological advances in personalized global positioning systems (GPS) have led to their increased use in capturing where people spend their time [7-12]. GPS technology in combination with measured physical activity levels have been used to identify the types of physical “built” environments associated with sedentary activities [13,14]. This technology has not been used frequently in longitudinal (or panel) studies of personal exposure to air pollution; instead these studies rely on traditional methods, such as time-activity diaries [15,16]. GPS technology could better identify microenvironments associated with the greatest exposures to pollutants [17-19], and indeed in a recent study which used GPS tracking through smartphones it was found to make a substantial difference in modeled estimates of exposures [20].

Within the context of a panel study of children with asthma in Montreal, Canada, we describe an automated classification of GPS data into location-based categories that makes use of temperature data to assist in discriminating indoor from outdoor locations. We compare our automated classification to time-activity diaries completed by the children. In addition, we compare continuous personal concentrations to fine particles (particulates with aerodynamic diameters 2.5 microns or less, PM_2.5_) assigned using both the time-activity diary and the automated GPS microenvironment data.

## Methods

The Montreal Asthma Panel Study was conducted in the eastern part of Montreal during the winter of 2009–2010 to determine associations between daily levels of personal exposures to selected air pollutants and acute symptoms and markers of respiratory and cardiovascular function.

Cohorts of up to six children were followed over a 10 day period. In total, 70 children participated in the study and carried a backpack that contained a GPS device and air pollution and temperature/relative humidity monitors. Participants were asked to take the backpack with them whenever they left home. However, if they were sitting or sleeping (i.e., in the home or at school), the backpack could be placed on a surface at breathing-level (not on the floor) in the same room.

The children also completed daily testing to measure markers of respiratory function and completed questionnaires about their physical home environment, current health and symptoms and recorded their locations and activities in a daily time-activity diary.

Ethics approval was obtained from research ethics boards at Health Canada, the McGill University Health Centre, the Direction de sante publique de Montreal, and Maisonneuve-Rosemont Hospital. Guardians provided written, informed consent for their child to participate in the study.

### Location of subjects from the GPS

Detailed data on location and speed (km/hr) were captured every second using a customized GPS unit (HeraLogger GPS) designed specifically for human time-activity studies. This unit has been well tested and provides an average precision of 7 m in typical urban conditions [21]. The GPS antenna was affixed to the strap of the backpack and pointed towards the sky to promote a strong signal. At the end of every sampling day, the data were downloaded to a personal computer. All data were subject to a quality control process that included removing points that were outside of the sampling date and time as recorded on the daily logsheets and removing points that the GPS device flagged as invalid. GPS points were excluded if the speed indicated by the GPS device exceeded 120 km/hr or if there were unrealistic deviations in the signal (>100 km over a few seconds).

### Geospatial datasets

In order to classify locations from the GPS, we obtained two geospatial vector datasets representing all building footprints in the study area (a polygon dataset from the City of Montreal) and roads (a line dataset from DMTI Spatial, CanMap Streetfiles). The participants’ home and school buildings were identified manually within the building dataset and saved as separate polygon datasets. For approximately 20% of the subjects’ homes, the available building dataset lacked footprints for the subjects’ homes or buildings which they visited. In these cases, each participant’s route was examined manually and compared to aerial maps obtained through Bing Maps (Microsoft Corporation) and a building footprint was manually added according to the aerial map outline.

### Personal air pollution measurements

Personal exposure to PM_2.5_ was measured using a personal DataRAM (pDR-1200; ThermoScientific, Waltham, MA) that was connected to a personal environmental monitor inlet (PEM; Chempass System R&P/Thermo) with a 37 mm Teflon after-filter. Sampling times were set at 1-minute intervals and the sampling rates were maintained with a pump that operated at 1.8 L/min. Personal measurements of concentrations were adjusted for any zero-drift based on pre- and post-sampling pump flows. The clock on the pDR was synchronized to the GPS onboard clock so that coincidental exposure and location values could be matched and analyzed with a high degree of temporal precision (<1 min). Air inlets for the pDRs were positioned in the backpack approximately in the breathing zone; for children who carried the backpacks, the inlets were attached to the shoulder strap and for the rolling backpack, the inlet was attached to the handle. The rolling backpack handle was close enough to a child’s face (<30 cm) to be considered within the breathing zone.

### Measurements of personal temperature and relative humidity

Each participant carried a small temperature and relative humidity monitor in an outside pocket of the sampling backpack (Hobo U10, Onset Computer Corp., Hoskin Scientific Ltd., Ontario) that recorded data every minute. We did not collect temperature for the first 13 subjects (130 sampling days), so these subjects were excluded from the present analysis.

### Self-reported time activity diaries

Participants recorded their daily activities in 30 minute increments in a diary format for every 24-hours of the sampling period (10 days total). Participants were asked to circle their location during that time-segment (“*Indoors-at-home”, “At home-in yard”, “At School”, “Indoors-away-from-home”, “Outdoors-away-from-home”, “In-transit”*) and to indicate activities in each 30-minute interval. “*At home-in yard”* and *“Outdoors-away-from-home”* were combined into one category that represented the outdoor environment. “At School” as referred to in the diary generally included all time, either indoors or outdoors, which took place at or near School. When students left the school building for recess, physical activity or lunch, most students did not note this outdoor time in their diary.

Although the children were asked to circle only one location, in three percent of the 30-minute increments participants circled two or more locations. We could not discern in what order multiple locations occurred (within the 30 minute interval). Instead of just assigning these entries as missing, we recoded them to a single location. The recoded location was selected from the multiple locations circled in the order of where people spend the most time. For example, if home was one of the multiple locations circled, then the recoded (single) location would be home. If home was not one of the options, then other locations were chosen in the following order: *“At School”, “Outdoors”, “Indoors-Other”* and *“In-transit”*.

### The automated classification method

The objective was to assign to each location in each 30-minute interval the corresponding latitude and longitude classified as “*Indoors-at-home”, “At School”, “Indoors-Other”, “Outdoors”, “In-transit”*. In theory, “mapping” the GPS data onto shapes representing buildings and roads appears simple. In practice, the GPS position often “drifts” around an actual location so that a person may appear to be outside their house even while they are at home. The approach that we developed used the recorded personal temperature to provide an indication as to the likelihood that a person was either indoors or outdoors. For example, if the temperature was consistently around 21°C and the time was 1 AM but the GPS signal showed outside of their home, the algorithm would reclassify the location as “*Indoors-at-home*”. Overall, this is a location classification method by GPS and temperature which was accomplished using Python scripting; we refer to this classification as our “automated” classification method.

Sampling days were included if there were at least 70% (approximately 17 hours) of non-missing, valid GPS points and complete diary data and 70% of complete personal temperature data during a 24-hour period. These conditions follow the often-used criteria used in air pollution sampling in which a 75% complete sample (hourly data) is acceptable for calculating a daily average. In our case, we chose to be slightly less restrictive by requiring 70% completion, as we were using shorter averaging periods (1 min, 30 mins) than a typical daily average.

**Figure 1:**
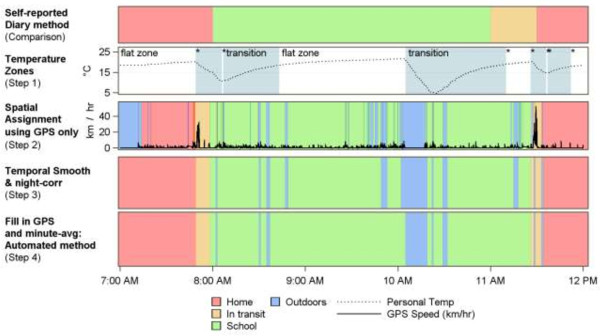
Steps of the automated GPS-based classification method.

The algorithm is described below and we provide an illustration in Figure 1.

### Step 1: temperature zone assignment

For each subject, two zones were identified: “transition” zones, where the temperature changed by more than 0.1°C per minute and “flat” zones where the temperature was relatively constant or changed very slowly. The temperature zones were created according to a detailed algorithm described in the Additional file 1.

### Step 2: spatial assignment

The GPS position (latitude and longitude), GPS speed (a speed reported by the GPS device), the time, and temperature zones were processed in ArcGIS (Esri, Redlands USA); using Python scripts to aid in bulk processing. First, all points were considered for “*In Transit*” assignment, regardless of zone. Second, different spatial classification approaches were used depending on whether the points were in a flat or in transition temperature zone (see Table 1).

**Table 1:** Step-wise approach for the spatial classification of GPS points

**What temperature zone is used?**	**Criteria for assigning points spatially compared to the base geospatial data (buildings, roads)? (in order)**	**What activity classification is assigned?**
None. This occurs **before** zones are considered (all points that fit the criteria are assigned)	Points with speed from the GPS >5 km/hr and within 20 m of a road segment	*In Transit*
Flat zone^1^ (temperature is relatively constant)	Points with GPS speed < 5 km/hr and within 25 m of road segment	*In Transit*
	Points within 25 m of subjects’ home	*Home*
	Points within 5 m of subjects’ school	*School*
	Points within 15 m of any other building	*Indoors Other*
	Points remaining (unclassified)	*Outdoors*
Transition zone (temperature is changing)	Points with GPS speed < 5 km/hr and within 5 m of road segment	*In Transit*
	Points within 5 m of subjects’ home	*Home*
	Points within 5 m of subjects’ school	*School*
	Points within 2 m of any other building	*Indoors Other*
	Points remaining (unclassified)	*Outdoors*

^**1**^Within flat zones, a spatial criterion was applied for all points in the zone, using only the location of the center (obtained using the “mean centers tool” in ArcGIS) point in the cluster.

#### Spatial classification of GPS data points (using temperature zones)

*The spatial classification of the GPS data proceeded as follows:*

a. All points with GPS speed >25 km/hr were classified “*In Transit*”, before zones were considered.

b. All points in each *flat zone* were averaged spatially using the “Mean Center” tool in ArcGIS. This created a single centroid point for all times in the flat zone. The centroid point was then classified according to Table 1 (for *flat zones).*

c. All remaining points were classified according to Table 1 (for *transition zones*).

### Step 3: temporal smoothing and night-time drift correction

We then applied smoothing to remove rapid shifts in location that were improbable and likely due to GPS drift not accounted for in the reclassification by temperature. For a 30-second moving window, we selected instances where the current classification (e.g., “*Outdoors*”) differed from the locations on both sides of the window of interest (e.g., “*Indoors-at-home*”). If the classification in the window differed (less than 30 seconds), we assumed that this was due to a short signal drift and we reclassified the window to be the same as its surrounding classifications.

Subsequently, we added steps to address signal drift at night. If a participants’ location had been classified as outdoors or in some other building within 100 m of their home between the hours of 11 pm and 5 am (within a flat temperature zone), these zones were then re-assigned as “*Indoors-at-home*”. As the asthmatic children were between 8 – 13 years of age we would expect them to be in bed.

### Step 4: averaging and filling in gaps in the GPS data: results in final automated classification

Gaps of less than two hours in the GPS data were filled-in as follows. If the locations on both sides of the gap were the same then the gap was “filled in” with the location surrounding the gap. Lastly, the per-second location data was summed for each minute and re-assigned to the most frequent location for that minute.

### Statistical analysis

The main objective of the analysis was to compare the physical locations of individual subjects assigned by the GPS-based automated classification method to their daily diaries. Further, we wanted to determine how differences between these two methods influenced estimated concentrations of PM_2.5_ from continuous personal measurements.

We excluded the 13 subjects for which measurements of temperature were not recorded. For sampling days that met the inclusion criteria, we calculated the daily percent time in each location (i.e., “*Indoors-at-home”, “At School”, “Indoors-Other”, “Outdoors”, “In-transit”*) as identified from the diary and the automated method. To compare with the self-reported diary method (which is in a 30 minute interval), we averaged the 1 minute automated data by selecting the most frequent location within each 30 minute interval. If there were 1-minute locations classified as “missing” within a 30 minute interval, then the most frequent non-missing location was used. In cases where all 30 minutes were missing, then the value remained coded as missing. Time-segments (both automated and diary) classified as missing were excluded from further analysis.

We estimated the concordance in each location between the reported daily diaries and the automated method by cross-classifying the six location categories. We computed the observed proportion of concordance by summing the proportions of agreement in each category. To account for chance, we made use of an agreement statistic, AC1 [22], that is similar to the multi-category Kappa statistic that is used commonly [7] but circumvents the known weakness of Kappa, being overly sensitive to trait prevalence and marginal probabilities. AC1 is interpreted in a manner similar to kappa: higher values of AC1 refer to a higher level of agreement. We computed the AC1 and the 95% confidence interval using a SAS macro [23]. In the Additional file 1: Table S2, we also present 2x2 concordances using the AC1 statistic and, for each location category, an estimated sensitivity and specificity which considers the diary-based method as an approximate “gold standard”.

**Figure 2:**
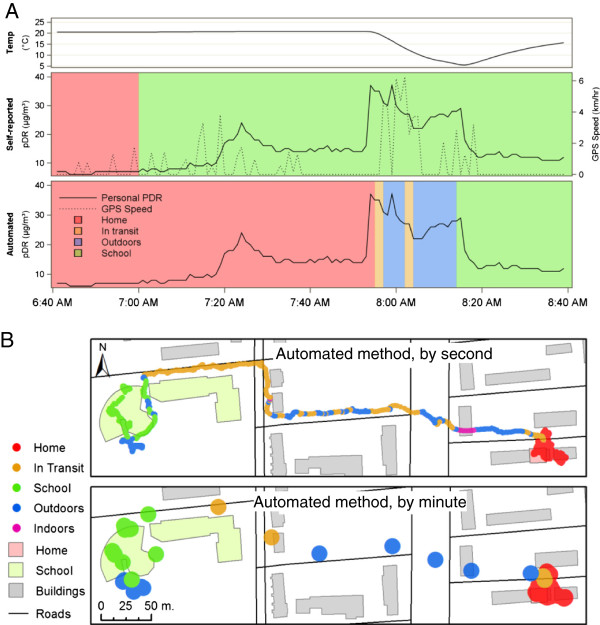
**Example of the impact of the automated classification system on personal exposure assessment. A)** The temporal component (temperature, GPS speed, and concentrations of PM2.5). **B)** The spatial component (classification of spatial locations).

Daily mean concentrations of PM_2.5_ were calculated for each sampling day for each subject using location categories as determined from the daily diaries and the (1 minute resolution) automated method, and these were then averaged across subjects. We also calculated the difference in mean concentrations between the two methods. We present an example where an individual’s morning particulate sampling data were plotted over their location classification categories according to both methods, see Figure 2A. This time-period was also mapped in GIS to demonstrate how the automated method captured geographic movement more precisely than the daily diary, see Figure 2B.

## Results

From 504 GPS files, we obtained 383 sampling days of participant data (54 participants of the total of 57) after limiting to only those meeting the inclusion criteria (>70% complete GPS and temperature data). Of the 121 excluded days, the average missing GPS data was 37%. After the automated classification method was applied, 547,372 (1-minute increments) and 18,383 (30 minutes) time-activity locations were available for analysis. At the 1-minute time-interval, 5,281 (1%) locations were missing diary data and 33,939 (6.2%) were missing GPS-based locations, leaving a total of 508,152 (1-minute) locations.

For the secondary analysis of the personal measurements of PM_2.5_, the dataset was further reduced to 293 sampling days (54 subjects) and 16,384 measurements. As the secondary analysis required air pollution measurements, a further 90 sampling days of data were excluded because of either missing or incomplete air monitoring data (caused usually by pump failure).

Table 2 shows selected characteristics of participants. There were no important differences in the study population demographics or time-activity diary data for the sampling days of all participants (n = 700) and those included in the analysis (n = 383).

**Table 2:** Selected Characteristics of Participants included in the present analysis, the Montreal Asthma Panel Study, 2009–2010

**Characteristics 54 participants)**	**Values**	**Frequency (%)**
Age	Mean (standard deviation)	9.6 (1.3)
	Minimum-maximum	8 – 13
*Race*	White	38 (70%)
	Black	9 ( 17%)
	Other	7 (13%)
*Gender*	Boys	41 (76%)
	Girls	13 (24%)
*Usual commute method to school*	On foot	24 (44%)
	Car, truck	17 (32%)
	Bus	13 (24%)
*Type of house or dwelling*	Detached house	17 (32%)
	Row house	8 (15%)
	Low rise apartment	10 (19%)
	High rise apartment	8 (15%)
	Du/Tri/Fourplex or Semi-detached	11 (20%)
*Year house built*	<1951	4 (7%)
	1951-1970	17 (31%)
	1971-2000	14 (26%)
	>2001	6 (11%)
	Did not know/no response	13 (24%)

Before presenting the main results, one particular example of how the daily diary could lead to misclassification that the automated method was able to correct is shown. Figure 2 shows a single morning (duration of two hours) for one participant where the child reported only “*Indoors-at-home*” and *“At School”* in their diary with no information about their *“In-transit”* to school. The concentrations of fine particles showed a distinct peak around 8 am (solid line on graph, shown in both self-reported and automated panels), thus suggesting that this exposure occurred during the transit to school. Using only the diary-based locations, this peak would be misclassified as occurring during school. Utilizing the GPS data and the automated location categories which incorporated temperature information, we obtained a more complete picture of this trip and these peaks are identified as occurring *“Outdoors”* and/or *“In-transit”*. Given the speed and travel pattern seen in the spatial map, we suspect that this child travelled by foot (walking) or bike to school leading to both *“Outdoors”* and *“In-transit”* being identified.

**Figure 3:**
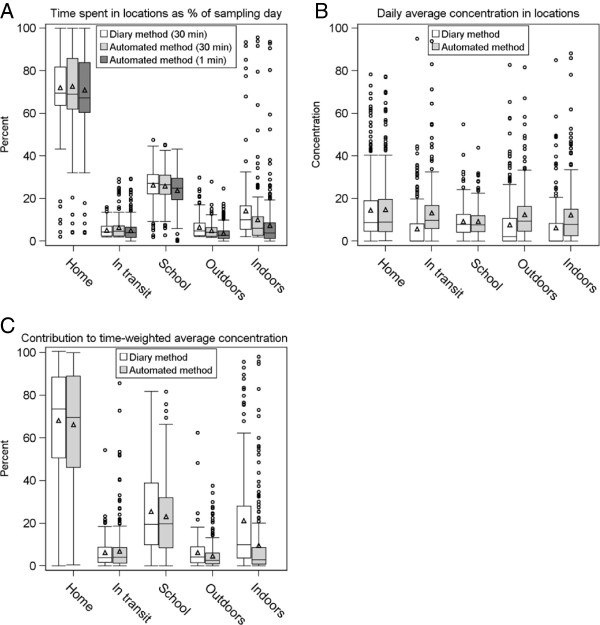
**Boxplots comparing diary to automated method A) Average daily percentage of time spent in locations (30 & 1 minute average). B)** Average daily PM2.5 concentration (μg/m3), not time-weighted **C)** Percent contribution (time-weighted) of average PM2.5 concentration to total daily average.

Figure 3A presents the distribution of the daily percentage of time spent in different locations (per sampling day) classified by the diary and automated methods. Except for “*Indoors-at-home*”, the results were similar when considering 30-minute increments. At 1-minute intervals, we observed larger differences, with the automated method estimating less time “*At School*”, “*Outdoors*” and “*Indoors-Other*. This is an important difference when considering acute exposures and health outcomes, especially when the locations being underestimated are typically “*In Transit*”.

Figure 3 (panels B and C) shows a comparison of mean concentrations and time-weighted concentrations of PM_2.5_ for the diary-based and automated methods according to location. (Additional results are presented in Additional file 1: Tables S3 & S4). A similar pattern was observed in Figure 3A for the mean percentage of time spent in each location. More striking differences were for locations where people spent less time for example, in which approximately 60% of the values assigned to “*In-Transit*”, “*Outdoors*” and “*Indoors-Other*” were different.

Table 3 presents a cross-classification of time-activity categories at 30-minute intervals between the diary-based and automated methods. The cells show the number and percentage (in parentheses) of 30-minute segments assigned to each category. The proportion of concordance between the diary-based and automated categories was 0.795 (95% confidence interval: 0.787- 0.780) and the chance-corrected agreement (Gwet’s AC1) [22] was 0.778 (95% confidence interval: 0.770-0.783). These results suggest “good” agreement between methods, over all location categories. Location categories such as Home showed relatively high levels of agreement (Additional file 1: Table S2: Home AC1 = 0.800; Additional file 1: Table S3: mean time spent at home is approximately 71% regardless of method) and the automated method had a sensitivity of 93.2% and specificity of 77.7% (Additional file 1: Table S2). However, location categories (“*Outdoors*” and “*In-Transit*”) where less time is spent show greater disagreement (Additional file 1: Table S3); for example, mean time “*Indoors-Other*” using the time-activity diary was 14.4% compared to 9.8% using the automated method, and the sensitivity (for Indoors-Other) was 28.8% but had a specificity of 98.8%. Note that in our analysis, this disagreement (as noted by agreement statistics and sensitivity/specificity) is likely inflated because we averaged the 1 minute automated method (GPS-based data) to a 30 minute interval for comparison with the diary-based method which would have removed many short duration events like “In-Transit” and “Outdoors” from the automated data prior to comparison.

**Table 3:** Cross-classification of the frequency (percentage %) of all sampling days comparing 30 minute time-location segments as reported by subjects in their daily diaries and as determined from the automated method

**Daily diary**^**1**^	**Automated method**						
	***Home***	***In transit***	***School***	***Outdoors***	***Indoors***	***Missing GPS***	***Total***
*Home*	11822 (64.3)	109 (0.6)	145 (0.8)	130 (0.7)	62 (0.3)	415 (2.3)	12683 (69)
*In transit*	145 (0.8)	91 (0.5)	43 (0.2)	40 (0.2)	30 (0.2)	52 (0.3)	401 (2.2)
*School*	230 (1.3)	92 (0.5)	2278 (12.4)	120 (0.7)	105 (0.6)	438 (2.4)	3263 (17.8)
*Outdoors*	298 (1.6)	39 (0.2)	123 (0.7)	98 (0.5)	48 (0. 3)	44 (0.2)	650 (3.5)
*Indoors*	398 (2.2)	141 (.8)	46 (0.3)	87 (0.5)	318 (1.7)	113 (0.6)	1103 (6.0)
*Missing diary*	199 (1.1)	6 (0)	5 (0)	13 (0.1)	9 (0)	51 (0. 3)	283 (1.5)
*Total*	13092 (71.2)	478 (2.6)	2640 (14.4)	488 (2.7)	572 (3.1)	1113 (6.1)	18383 (100)

^1^*Proportion of concordance observed (P*_*o*_*) = 0.795 (95% confidence interval: 0.787, 0.800).*

Agreement statistic: Gwet’s AC1 = 0.778 (95% confidence interval: 0.770, 0.783).

Additionally, these results are further reflected in the mean PM_2.5_ data within each location category (Additional file 1: Table S4 and Figure S2). The scatterplots for the PM_2.5_ data illustrate good correlations for the mean daily “*Indoors-at-home*” concentrations regardless of which categorization method is used, perhaps because potentially misclassified peaks of exposure are relatively unimportant to overall averages and are “washed out” due to the longer time spent at home. By contrast for the “*In-Transit*”, “*Indoors-Other*” and “*Outdoors*” locations, there is a group of points which are assigned “0” on the self-reported classification but had non-zero concentrations using the automated method, suggesting that the diary-based method is missing these exposures completely. These findings contrast with results identified in the review by Kelly et al., [24] where eight studies were shown to over predict journey times using diary-based methods compared to the GPS method. The differences that were identified in the review suggest that the diary data were between +2.2 to +13.5 minutes greater than the GPS data. Wu et al. [12] also indicated that pregnant women overestimated their time spent in transit when comparing diary-based data with GPS data.

## Discussion

We developed a method for classifying personal GPS route data in order to generate location-activity based categories that could conceivably replace standard diary-based methods. This builds on work by Mavoa et al. [11], who developed a sequence alignment method to match GPS and diary data. They were able to demonstrate that an automated approach could explain approximately 62% of the children’s activities compared to conducting a manual matching approach which explained approximately 70%, but was an order of magnitude more time intensive. Wu et al. [25], also utilized GPS data to assign locations to free-living individuals in California. They were able to compare two models with activity logs to assess the feasibility of using just GPS data to assign locations including indoor, outdoor – static, outdoor – walking and in-vehicle. Their models functioned well for indoor and in-vehicle locations suggesting that batch processing for these environments may be feasible. Our use of temperature in conjunction with the GPS data is novel and addresses one of the challenges from using GPS data alone to classify locations. This approach has allowed us to classify GPS data for specific locations (home and school) with greater accuracy. We applied the automated classification to GPS routes and concentrations of PM_2.5_, and compared the results to diary-based classifications.

For the most part, the key differences in concentrations of PM_2.5_ and time spent according to the two methods were for “*In-Transit*”, “*Outdoors*” and “*Indoors-other*” locations. Given that these environments represent shorter exposure windows and therefore presents more of a challenge for accurate reporting, the strength of the GPS method may be that it better reflects these short-duration events especially when using diaries with relatively large windows of reporting time. Specifically, self-reported diaries may be inaccurate for these short duration events.

Others [12,24] have shown that diary-based methods over-predicted travel time when compared to various GPS-based approaches. This suggests that self-reported transit data are less accurate than GPS methods which is important as these locations can also be responsible for some of our peak exposures to PM_2.5_[6]. In a study of pregnant women Wu et al. [12] estimated exposures to polycyclic aromatic hydrocarbons based on time spent in transit using self-reported and GPS data and using a previously developed regression-based model for PAH concentrations. They found, on average, there was an overestimated exposure of 15.8% using the self-reported diaries. Our study is one of the first to combine direct GPS-based classifications with continuous air pollution monitoring of fine particulate matter (personal measurements) in a real study population.

There are some characteristics of our automated GPS-based approach which could limit its use. This method required collecting personal temperature data and utilized the strong temperature differences between indoors and outdoors that often occur in Montreal during the winter. This may be feasible in hotter climates where air conditioning is commonly used indoors but could be less useful in climates where milder meteorological conditions are found and window opening is typical or in (e.g. fall-spring) seasons where temperature varies less from indoors to outdoors. Kim et al. [26] recently reported a method for assessing time spent indoors using GPS data. The authors were able to identify the number of available satellites being picked up by the GPS and when less than 9 satellites were picked up for a minimum of 3 minutes the location was determined as indoors, this may be a suitable alternative for climates where the temperature differences between indoor and outdoor environments are not as extreme as in Montreal. Another study of children’s location assessed the combination of GPS and light sensors, the authors were able to distinguish with moderate to high levels of accuracy whether the children were indoors or outdoors based on the differences in light intensity in the two locations, there were potential limitations in climates with cloudy conditions [27]. There were also some issues where the GPS signal was missing and data needed to be interpolated; while interpolations were limited to only short time periods (where the locations on either side of the gap were the same), some short trips may have been missed.

The data processing method also reduced the impact of GPS point drift, a phenomena where the GPS-recorded position deviates randomly around a true location, likely caused by changes in the satellite configuration. This is a problem that others have identified [7,25] as being of concern especially when inside or transitioning between different building structures. Resolving this issue is a challenge for designers of wearable, long-lasting GPS for capturing personal mobility.

Other studies that have included a GPS as part of their exposure assessments have found that compliance is generally good [7,11,18,19] with the most common complaint being that participants forget to bring the GPS with them on trips. This issue is relevant to personal air pollution monitoring studies: if participants fail to bring their monitoring equipment with them as they move between microenvironments it will never be possible to accurately capture exposures. Combining GPS with personal air pollution monitoring helps to determine if participants’ did bring equipment with them on all trips which were indicated in their self-reported diary.

Other investigators who have manually and automatically classified GPS data have found that self-reported diaries were inaccurate when compared with GPS data, specifically, with the majority of individuals underestimating time spent in some locations (e.g. “*Outdoors*”, or “*In–Transit*”) or doing specific activities [9,11,25,28,29]. Generally, in this study, there was good agreement between self-reported and automated time-activity categorizations (which are based on GPS, but incorporated temperature as well). Similar to other studies, it was determined that children generally under reported “*In-Transit*” and “*Outdoors*” activities, and agreement was strongest for categories where most time is spent, specifically, “*Indoors-at-Home*” and “*At-School*”. Wu et al. [25] reported sensitivities and specificities (indoor: sensitivity 85-95%, specificity ~82%) comparing modeled estimates to a true “gold standard” (participant diaries validated and corrected by GPS, GIS and detailed interviews); our results for home (93% and 78%), and school are comparable (70% and 97%), but our approach did not perform as well for other indoor locations. However, our diary-based “gold standard”, is not validated by interviews and likely biased by under reporting of locations with smaller time periods. With a recent focus on distinguishing between ambient (generally outdoor) and non-ambient exposures to PM_2.5_[17,30-33], having access to GPS-based locations will assist in separating these exposures more accurately. This study demonstrated how inaccurate time-activity classification could lead to exposure misclassification, specifically in locations where people are spending less time (e.g. “*In-Transit*” and “*Outdoors*”).

## Conclusion

The results of the study demonstrate the successful development and application of an automated time-activity diary approach that generated time-activity classifications based on GPS and temperature data alone. By comparing the automated approach to a diary-based method, there was good agreement with most classifications, and some of the normal weaknesses of the diary-based approach specifically in capturing short-duration events were improved. The analysis indicates that the automated approach better captures locations that may produce important sources of exposure.

## Abbreviations

GPS: Global Positioning Systems; pDR: Personal DataRAM.

## Competing interests

The authors know of no competing interests either financial or otherwise.

## Authors’ contributions

EN was responsible for the data management, data analyses and interpretation. She also wrote the manuscript. GM was responsible for managing the data collection and contributed to the writing of the manuscript. DR designed the GPS and software, provided guidance on data collection and data interpretation. He also contributed to the writing of the manuscript. MG was the co-Principal Investigator for the study, responsible for the study design, data collection, statistical interpretation, and contributed to the writing of the manuscript. AW was the co-Principal Investigator for the study, responsible for the study design, data collection, data management, and contributed to the writing of the manuscript. All authors read and approved the final manuscript.

## Supplementary Material

Additional file 1: Table S1 2×2 Cross-classification of the frequency (percent of total) time spent in different locations comparing the automated and self-reported diary methods at 30 minute intervals; used to obtain results in **Table S2**. **Table S2** Concordance results and sensitivity, specificity and positive predictive value (PPV) (assuming the Diary as a “gold standard”) from 2×2 cross-classifications of the self-reported diaries and automated methods (30 minute intervals), n=18383 location pairs. **Table S3** Percent of day spent in different locations by classification method. **Figure S1** Daily time spent in locations comparing the automated GPS-based and self-report methods. **Figure S2** Daily average concentrations of PM2.5 (μg/m3) comparing the automated GPS-based and self-report methods 1. Click here for file
