# Supplementary material for: Using Global Positioning Systems (GPS) and temperature data to generate time-activity classifications for estimating personal exposure in air monitoring studies: an automated method
Source: Environ Health. 2014 May 8;13:33. doi: 10.1186/1476-069X-13-33 (PMC4046178; doi:10.1186/1476-069X-13-33)
Supplement: Additional file 1: Table S1 — 2×2 Cross-classification of the frequency (percent of total) time spent in different locations comparing the automated and self-reported diary methods at 30 minute intervals; used to obtain results in Table S2. Table S2 Concordance results and sensitivity, specificity and positive predictive value (PPV) (assuming the Diary as a “gold standard”) from 2×2 cross-classifications of the self-reported diaries and automated methods (30 minute intervals), n=18383 location pairs. Table S3 Percent of day spent in different locations by classification method. Figure S1 Daily time spent in locations comparing the automated GPS-based and self-report methods. Figure S2 Daily average concentrations of PM2.5 (μg/m3) comparing the automated GPS-based and self-report methods 1. [file 1476-069X-13-33-S1.docx]

**Additional File 1: Tables & Figures**

| **Diary method** | **Automated method** | |
| --- | --- | --- |
|  | *Home* | *Other* |
| *Home* | 11822 (64.3) | 861 (4.7) |
| *Other* | 1270 (6.9) | 4430 (24.1) |

| **Diary method** | **Automated method** | |
| --- | --- | --- |
|  | *Transit* | *Other* |
| *Transit* | 91 (0.5) | 310 (1.7) |
| *Other* | 387 (2.1) | 17595 (95.7) |

| **Diary method** | **Automated method** | |
| --- | --- | --- |
|  | *School* | *Other* |
| *School* | 2278 (12.4) | 985 (5.4) |
| *Other* | 362 (2) | 14758 (80.3) |

| **Diary method** | **Automated method** | |
| --- | --- | --- |
|  | Outdoors | Other |
| *Outdoors* | 98 (0.5) | 552 (3) |
| *Other* | 390 (2.1) | 17343 (94.3) |

| **Diary method** | **Automated method** | |
| --- | --- | --- |
|  | Indoors | Other |
| *Indoors* | 318 (1.7) | 785 (4.3) |
| *Other* | 254 (1.4) | 17026 (92.6) |

Table S1

2x2 Cross-classification of the frequency (percent of total) time spent in different locations comparing the automated and self-reported diary methods at 30 minute intervals; used to obtain results in Table S2

|  | **Proportion of concordance (observed)** | **AC1  (95% CI)** | ***Sensitivity^1^*** | ***Specificity^2^*** | ***Precision^3^*** |
| --- | --- | --- | --- | --- | --- |
| **Home** | 0.884 | 0.800 (0.79, 0.81) | 93.2% | 77.7% | 90.3% |
| **Transit** | 0.962 | 0.960 (0.96, 0.96) | 22.7% | 97.8% | 19.0% |
| **School** | 0.929 | 0.900 (0.89, 0.91) | 69.8% | 97.6% | 86.3% |
| **Outdoors** | 0.934 | 0.945 (0.94, 0.95) | 15.1% | 97.8% | 20.1% |
| **Indoors Other** | 0.936 | 0.938 (0.93, 0.94) | 28.8% | 98.5% | 55.6% |

Table S2 Concordance results and sensitivity, specificity and positive predictive value (PPV) (assuming the Diary as a “gold standard”) from 2x2 cross-classifications of the self-reported diaries and automated methods (30 minute intervals), n=18383 location pairs

1 Sensitivity was calculated as the true (both methods) positive estimated/(true positives + false negative estimates)

2 Specificity was calculated as the true (both methods) negatives estimated/(true negatives +false positive estimates)

3 Precision was calculated as the true (both methods) positives/(true positives + false positive estimates)

| ***Locations*** | ***Diary method  (30 min)*** | | ***Automated method  (30 min)*** | | ***Automated method  (1 min)*** | |
| --- | --- | --- | --- | --- | --- | --- |
|  | ***N*** | ***Mean %time(SD)*** | ***N*** | ***Mean %time(SD)*** | ***N*** | ***Mean %time(SD)*** |
| Home | 383 | 71.4% (14.9) | 383 | 71.7% (16.7) | 383 | 70.9% (16.9) |
| In transit | 159 | 5.4% (3.2) | 229 | 6.5% (5.3) | 365 | 5.0% (4.5) |
| School | 243 | 26.9% (7.2) | 240 | 26.2% (7.2) | 246 | 23.9% (8.3) |
| Outdoors | 206 | 6.7% (5.2) | 202 | 5.1% (3.7) | 351 | 3.7% (3.5) |
| Indoors | 153 | 14.4% (15.9) | 206 | 9.8% (14.0) | 288 | 7.4% (12.4) |
| Missing | 101 |  | 300 |  | 229 |  |

Table S3 Percent of day spent in different locations by classification method

| Time activity classification | N | Mean daily Concentrations of PM_2.5_  (Standard deviation) µg/m^3^ | | | % of values greater than a difference of 5 µg/m^3^ between methods |
| --- | --- | --- | --- | --- | --- |
|  |  | Diary  method  (30 min) | Automated  method  (30 min) | Mean  Difference |  |
| Home | 292 | 17.1 (25.1) | 17.0 (23.0) | -0.1 (8.2) | 4% |
| In transit | 284 | 6.8 (19.3) | 15.9 (29.0) | 9.1 (32.0) | 61% |
| School | 202 | 9.3 (7.5) | 9.3 (6.9) | 0.0 (4.5) | 5% |
| Outdoors | 276 | 9.6 (25.1) | 14.3 (33.1) | 4.7 (38.9) | 58% |
| Indoors | 236 | 10.4 (29.7) | 15.9 (28.8) | 5.5 (36.3) | 56% |

Table S4 Personal fine particulate concentrations (µg/m^3^) for each sampling day, averaged across the time activity diary classifications

| 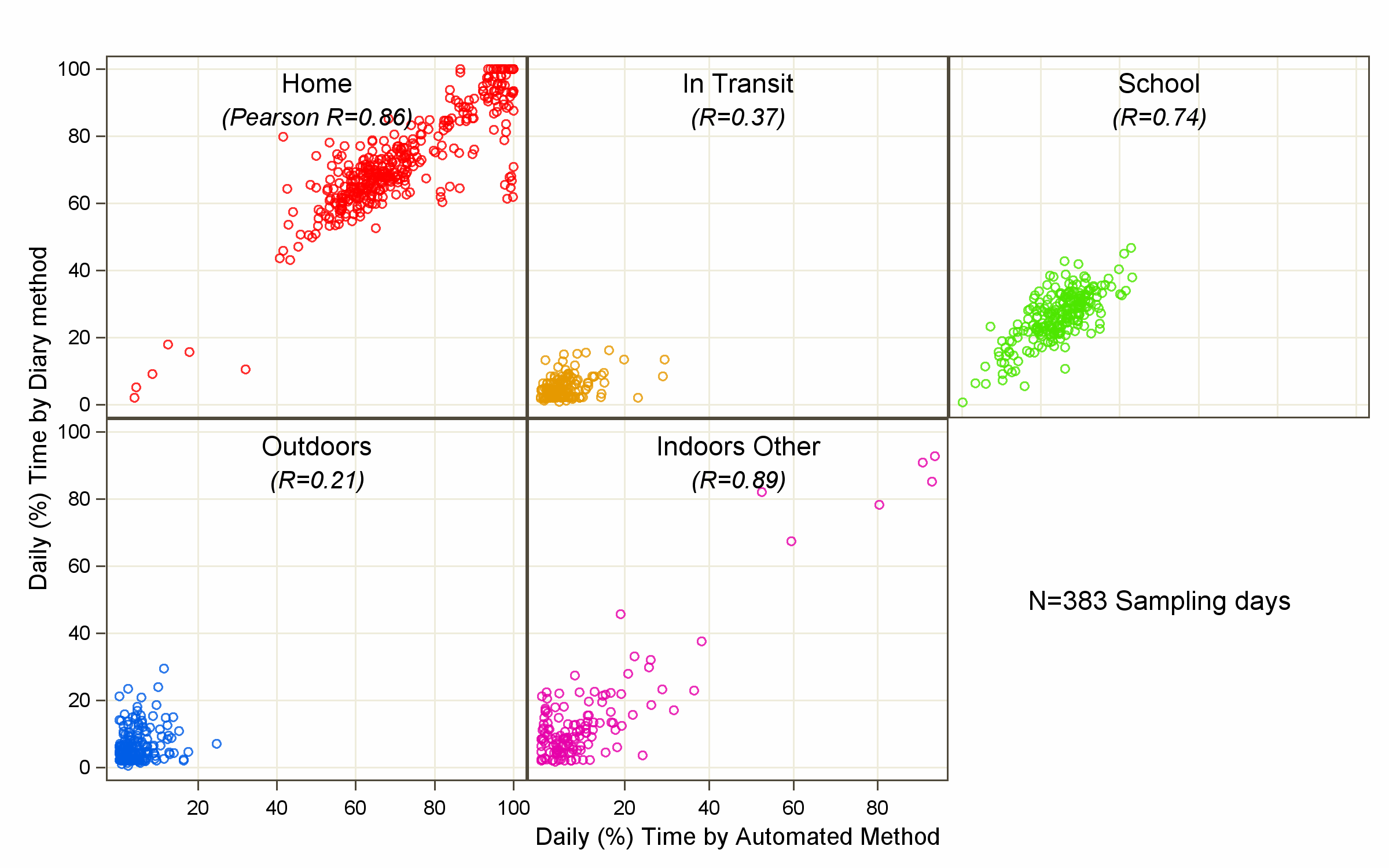 |
| --- |
| Figure S1 Daily time spent in locations comparing the automated GPS-based and self-report methods. |

|  |
| --- |
| 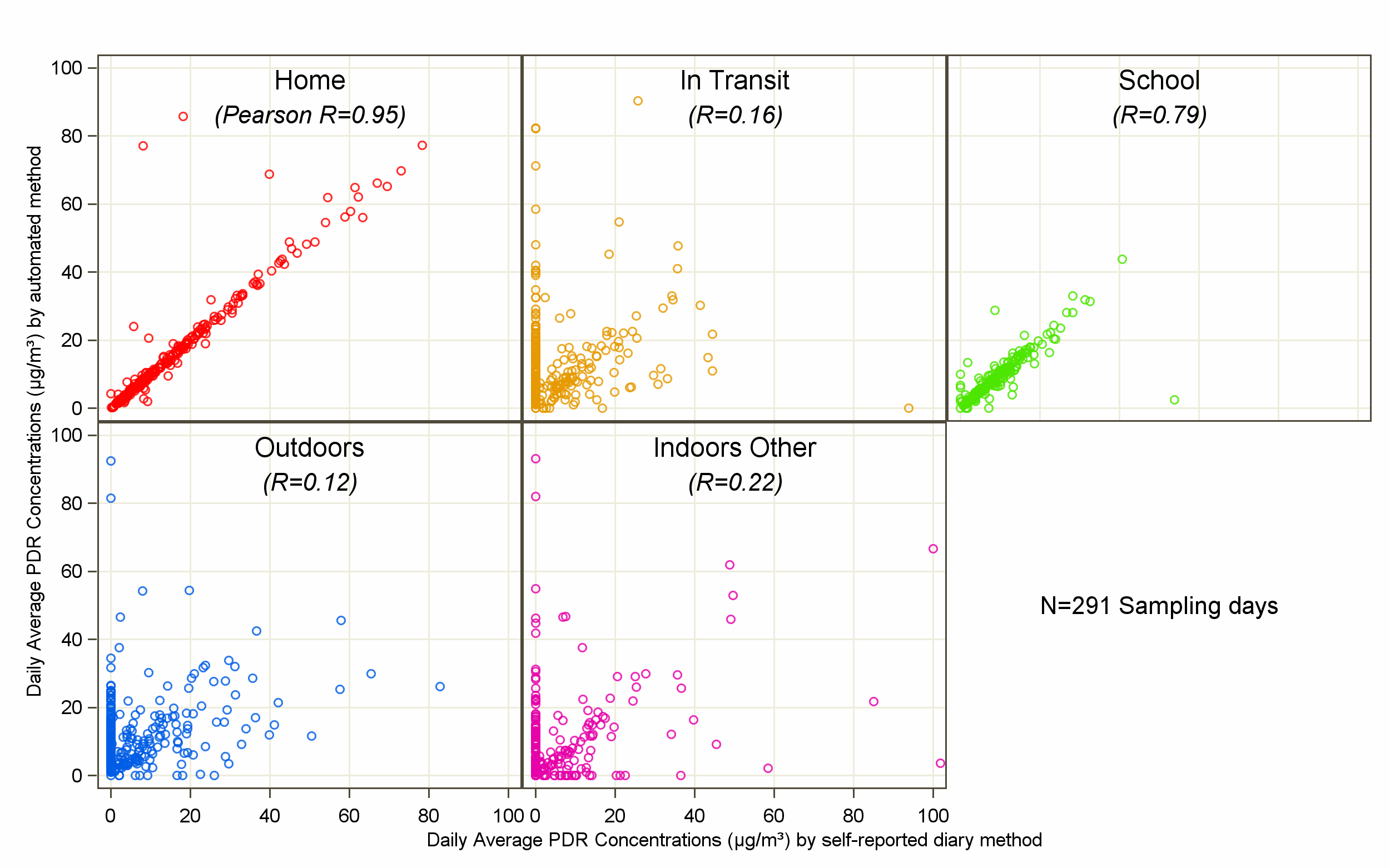  Figure S2 Daily average concentrations of PM_2.5_ (μg/m^3^) comparing the automated GPS-based and self-report methods. ^1^  1 Cases where concentration (based on diary method) is 0 represent situations where exposure was assigned in a different category than the automated method present an example of possible exposure misclassification. |

**Additional File 1: Text**

**Detailed description of the temperature zone assignment (step 1)**

For each subjects’ temperature data, two zones were identified: “transition” zones, where the temperature changed by more than 0.1°C per minute and “flat” zones where the temperature only changed very slowly (less than 0.1°C per minute). The temperature zones were created according to a detailed algorithm described here.

1. We calculated the difference between each temperature measurement and the previous measurement (1 minute difference)
2. We calculated a moving average of the previous, current and next temperature difference
3. If the moving average >0.1°C then we assigned the location as a “transition”; otherwise it was defined as a “flat zone”
4. If current zone was classified as a transition *but* the prior and subsequent zone were both flat, then we changed the current zone to flat. This removed instances where a slight increase in slope during a long flat segment is classified incorrectly as a transition zone.
5. To identify cases where the temperature was changing rapidly (usually decreasing) and then very briefly (<2-3 minutes) stabilized before changing rapidly again (usually increasing). If the current zone was classified as a transition *but* the slope (from a.) was <0.1°C and the difference between the current moving avg (b.) and prior minutes’ moving avg (b_lag) was > 0.1°C then the zone was changed to flat.
